# Prevalence of Haemosporidian Parasites in an Arctic Breeding Seabird Species—The Red-Throated Diver (*Gavia stellata*)

**DOI:** 10.3390/microorganisms10112147

**Published:** 2022-10-29

**Authors:** Birgit Kleinschmidt, Monika Dorsch, Stefan Heinänen, Julius Morkūnas, Yvonne R. Schumm, Ramūnas Žydelis, Petra Quillfeldt

**Affiliations:** 1 Department of Animal Ecology and Systematics, Justus Liebig University Giessen, 35392 Giessen, Germany; 2 BioConsult SH, 25813 Husum, Germany; 3 DHI, 2970 Hørsholm, Denmark; 4 Raasepori Campus (Raseborg), Novia University of Applied Sciences, Raseborgsvägen 9, 10600 Ekenäs, Finland; 5 Marine Research Institute, Klaipėda University, 92294 Klaipėda, Lithuania; 6 Ornitela UAB, 03228 Vilnius, Lithuania

**Keywords:** avian malaria, Leucocytozoon, Plasmodium, GAVSTE01, GAVSTE02

## Abstract

Haemosporida, vector-transmitted blood parasites, can have various effects and may also exert selection pressures on their hosts. In this study we analyse the presence of Haemosporida in a previously unstudied migratory seabird species, the red-throated diver *Gavia stellata*. Red-throated divers were sampled during winter and spring in the eastern German Bight (North Sea). We used molecular methods and data from a related tracking study to reveal (i) if red-throated divers are infected with Haemosporida of the genera *Leucocytozoon, Plasmodium* and *Haemoproteus*, and (ii) how infection and prevalence are linked with the breeding regions of infected individuals. Divers in this study were assigned to western Palearctic breeding grounds, namely Greenland, Svalbard, Norway and Arctic Russia. We found a prevalence of *Leucocytozoon* of 11.0% in all birds sampled (n = 45), of 33.0% in birds breeding in Norway (n = 3) and of 8.3% in birds breeding in Arctic Russia (n = 25). For two birds that were infected no breeding regions could be assigned. We identified two previously unknown lineages, one each of *Plasmodium* and *Leucocytozoon*. Haemosporida have not been detected in birds from Greenland (n = 2) and Svalbard (n = 2). In summary, this study presents the first record of Haemosporida in red-throated divers and reports a new lineage of each, *Plasmodium* and *Leucocytozoon* GAVSTE01 and GAVSTE02, respectively.

## 1. Introduction

Birds are frequently infected with a number of intracellular blood parasites, including Haemosporida, or hematozoa, of the genera *Plasmodium, Haemoproteus* and *Leucocytozoon* [1,2,3,4]. *Plasmodium, Haemoproteus* and *Leucocytozoon* are vector-borne parasites that cause avian malaria or malaria-like disease in birds [5,6,7]. However, there are interspecific differences in the prevalence of haemosporidian parasites and seabirds mostly have low levels or even a complete lack of infections [8]. The most common haemosporidian parasites found in seabirds were the genera *Haemoproteus* and *Plasmodium*, each showing infections for 13% of the species studied [8]. In Antarctic regions Haemosporida were absent from seabirds [8], whereas in Arctic regions few records are known so far [9,10]. In the context of climate warming the prevalence and distribution of Haemosporida may change, particularly in high latitude areas [10,11,12,13] and some of the dipteran vectors are quite common in arctic regions. These vectors are mosquitos (Culicidae) which transmit *Plasmodium*, louse flies (Hippoboscidae) and biting midges (Ceratopogonidae) which transmit *Haemoproteus*, and blackflies (Simuliidae) which transmit *Leucocytozoon* [14].

In general, haemosporidian parasites are considered pathogenically low in bird populations [15], but in songbirds and waterfowl, deaths, population declines and extinctions are related to Haemosporida infestations. [16,17,18]. Reduced body condition, expression of sexual ornaments, as well as lower reproductive success of hosts has been associated with infection [19,20,21,22,23]. Thus, haemosporidian parasites can affect survival and reproduction, exerting significant selection pressure on their hosts [24,25,26,27]. A co-infection with more than one lineage even increases the risk of reduced body condition, caused by the additive cost from single to double infection [28]. In this context, Hegeman et al. [29] found that a haemosporidian infection affects migratory birds’ movements by prolonging their stop-over duration.

Our study species, the red-throated diver *Gavia stellata* is a migratory Arctic-breeding seabird species for which there have been no known studies of haemosporidian parasites, to our knowledge. *Gavia stellata*, colloquially known as the red-throated loon in America or the red-throated diver in Europe, has a Holarctic breeding distribution, inhabiting small lochs adjacent to coastal areas. Inland areas are rarely occupied during the breeding season as this species prefers to feed in marine habitats [30]. Red-throated divers spend the non-breeding season in the Atlantic Ocean, North- and Baltic Sea and transmission of blood parasite infections via biting insects is thus limited to the breeding season. This seabird species is affected by increasing anthropogenic impacts, such as shipping traffic and offshore wind turbines in its non-breeding areas, to which this species reacts very sensitively [31,32,33,34]. In this context, infestation with Haemosporida could have an additional negative effect and information about the presence or absence of these parasites is an important basis when considering cumulative effects. Within the Gaviidae family, an infection with Haemosporida has so far only been documented for great northern divers (common loons, *Gavia immer*), with infections of *Plasmodium* as well as *Leucocytozoon* [35,36,37].

We took blood samples from 45 red-throated divers captured between 54° N 7° E and 55° N 8° E in an internationally important non-breeding habitat, the eastern German Bight (North Sea) in winter and spring within the framework of the DIVER project [31,38,39,40,41,42]. The DIVER project is a tracking study, and therefore information on the breeding areas in Greenland (−20° W 76° N; −50° W 69° N), Svalbard (16° E 76° N; 15° E 77° N), Norway (8° E 60° N–25° E 70° N) and northern Russia (41° E 66° N–103° E 73° N) of single individuals was available to be linked with haemosporidian parasite infection. The overall objective of the study presented here is to document the infestation of haemosporidian parasites in European red-throated divers. This information can be considered when assessing habitat change and thereby a possibly increased stress level in their European non-breeding habitats. Specifically, we aim to (i) present general information about the presence/absence of the three haemosporidian genera *Haemoproteus, Plasmodium* and *Leucocytozoon*, (ii) to study prevalence and (iii) lineage richness among the sampled red-throated divers.

## 2. Materials and Methods

### 2.1. Sampling and Sample Preparation

Bird capture and sampling were carried out in accordance with the local legislation. Sampling was conducted in the eastern German Bight (North Sea Germany) about 20 to 30 km west of the island of Amrum in three consecutive years: March to April 2015, February to March 2016 and March 2017 [31,38,39,40,41,42]. A total of 45 red throated divers were captured to be tagged with satellite transmitters within the study area. Birds were captured at night, from a RIB (rigid inflatable boat) using a hand net and the “night lighting technique” [43,44]. Blood samples were taken with a sterile needle and stored on FTA cards (Whatman FTA card technology, Sigma Aldrich, Darmstadt, Germany) for further analysis. In the laboratory, a 2 × 2 mm piece of the dried blood sample was cut out of the FTA card and the DNA was isolated using an ammonium acetate protocol adapted from Martinez et al. [38,45] and purified with NZYGelpure columns (NZYTech, Lisbon, Portugal). A NanoDrop2000c UV-Vis spectrophotometer (Thermo Fisher Scientific, Wilmington, NC, USA) was used to determine the final DNA concentration of the sample and the extracted DNA was stored frozen until further analysis.

### 2.2. Analyzing the Presence-Absence, Prevalence and Lineage Richness of Haemosporida with Molecular Tools (Nested PCR and Sanger Sequencing)

The measures of parasitism can be distinguished as prevalence, which refers to the proportion of individuals that are infected, parasitemia, which refers to the number of infected blood cells or the density of parasites within infected hosts, and richness, which refers to the number of parasite species/lineages found in an individual host, a group, or a species [46,47].

Parasitemia was not examined due to the absence of blood smears. Presence–absence data of Haemosporida (*Haemoproteus, Plasmodium* and *Leucocytozoon*) was studied using the blood samples (n = 45), which were screened with a nested polymerase chain reaction (PCR) targeting a 479 bp region of the cyt b gene [48]. The nested PCR protocol corresponds to a two-step PCR which allows simultaneous typing of species from the three most common avian blood parasite genera (*Haemoproteus, Plasmodium* and *Leucocytozoon*) [48]. First, an initial PCR step was applied using the primer combination HaemNFI/HaemNR3. Second, a 4 µL aliquot of this PCR product was subsequently used as a template to specifically detect *Haemoproteus* and *Plasmodium* using the primer combination HaemF/HaemR2 or *Leucocytozoon* using the primer combination HaemFL/HaemR2L (Table 1). The three PCR reactions were each set in a 25µL reaction volume that contained 12.5 µL 2× Dream Taq PCR Master Mix ready-to-use solution (Thermo Fisher Scientific, Germany), 4 µL of template DNA, 0.6 µM of each primer (1.65 µL of 10 µM) and sterile water. DNA from passerine birds with a known infection served as a positive control and deionized water as a negative control. Cycling conditions followed the protocol given by Hellgren et al. [48]. An incubation step at 94 °C for 3 min, a final extension at 72 °C for 10 min and a thermal profile of 30 s at 94 °C, 30 s at 50 °C and 45 s at 72 °C for 20 cycles was applied in the initial PCR, and for 35 cycles in the parasite-specific PCR. PCR protocols were carried out on a Biometra TOne Cycler (Analytik Jena, Jena, Germany). All samples were screened twice using the same protocol to back up positive or negative results.

PCR amplicons were visualised using high-resolution capillary gel electrophoresis (QIAxcel Advanced, Qiagen, Bern, Switzerland). Samples that showed a distinct peak (478/480 bp) were bi-directional Sanger sequenced using a Microsynth-Seqlab (Sequence Laboratories, Goettingen, Germany). Forward and reverse sequences were assembled and trimmed in a CLC Main Workbench 7.6.4 (CLC Bio, Qiagen, Denmark). Sequences were aligned to reference sequences deposited in the MalAvi database [2] and GenBank nucleotide databases using BLASTN 2.3.0+ [49] to identify lineages. If the sequences differed by one or more nucleotides in the cyt b fragment they were considered as distinct lineages [2,48,50]. Lineages that were considered as distinct and had no database records in MalAvi were considered novel and were named according to MalAvi nomenclature.

### 2.3. Phylogenetic Analyses

The best matching Malavi lineage and the best associated GenBank entry were downloaded as respective reference sequences. The downloaded reference sequences (Table 2), the consensus sequences from our own samples and a sequence from Babesia [GenBank record KC754965 [51]] as the outgroup were aligned in BIOEDIT using the ClustalW multiple alignment tool. Ten nucleotide sequences were considered in the final alignment (one outgroup, four sequences from this study and five reference sequences). jModelTest 2.1.10 and Bayesian Information Criterion scores were used to select the best suitable nucleotide substitution model (HKY) for our alignment. BEAST v1.8.4. was used to generate a Bayesian phylogenetic tree. In BEAUTi v1.8.4 model parameters for this analysis were selected with the HKY substitution model, strict clock as clock type and a Yule speciation process as tree prior. The chain length for the Metropolis coupled Markov Chain (MCMC) was set to 25 Mio. generations (burn-in 10%), and one tree was recorded every 1000 generations. We verified the trace for convergence using Tracer v1.6. TreeAnnotator in BEAST v1.8.4 was used to generate a maximum clade credibility tree (MCCT). Finally, FigTree v1.4.3 [52] was used to visualize the final phylogenetic tree. The similarities between sample sequences were calculated in BLAST [53].

## 3. Results

### 3.1. Presence–Absence, Prevalence and Parasite Richness

Using molecular methods we screened 45 samples for haemosporidian parasites. Samples that were screened positive (n = 5) were sequenced successfully and haemosporidian genera, as well as lineages, could be assigned. Two samples were excluded from the phylogenetic tree reconstruction due to insufficient sequence length. Haemosporidian parasites were present in five individuals resulting in an overall prevalence of 11.0% in all screened red-throated divers in this data set. Of the five infected individuals all were infected with a single *Leucocytozoon* lineage and one of these five individuals showed a heterogenic infection (co-occurrence of different parasite genera) with *Leucocytozoon/Plasmodium*. *Haemoproteus* infections were not detected in this sample set. Detailed information on the positively tested individuals and the sample location can be found in Table A2 and Table A3.

### 3.2. Lineage Diversity

**Figure 1:**
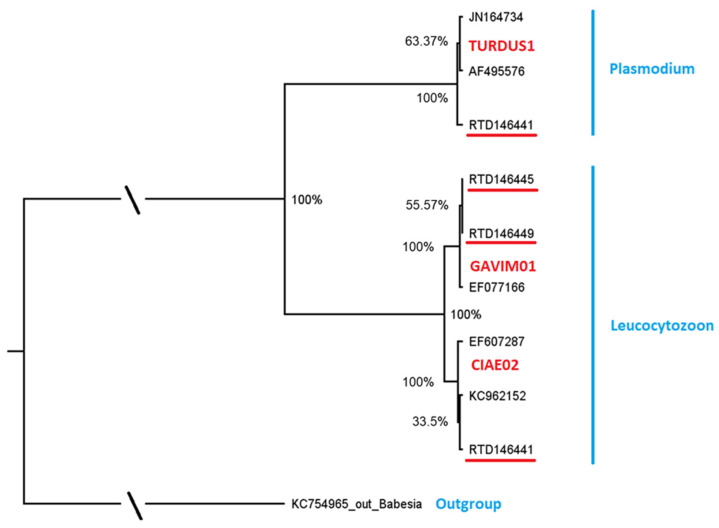
Molecular phylogenetic analysis by Maximum Likelihood Bayesian Analysis, based on parasite DNA sequences (479 bp cyt b fragment). Posterior probabilities of the nodes are shown. Genus names of parasites are indicated in blue, MalAvi lineage names are included in red letters and parasites from red-throated diver samples are given with bird IDs, indicated in black and underlined red. Reference sequences are indicated in black and not underlined. Details of the reference sequences, including blood parasite and host species identity and code and location, are given in Table 2.

We found a comparatively high lineage diversity in the five infected individuals. *Leucocytozoon* was present with three lineages and *Plasmodium* with one lineage resulting in a lineage richness of 8.8% (Figure 1, Table 3).

The MalAvi database [MalAvi, 2] was used to determine if the encountered lineages had previously been found in other bird species. One Plasmodium lineage GAVSTE01 (n = 1) and the most prevalent Leucocytozoon lineage GAVSTE02 (n = 2) each differed by one nucleotide in the cyt b fragment from lineages reported previously and were considered as distinct and novel. Detailed information on novel sequences can be found in Table A1. The novel Leucocytozoon lineage GAVSTE02 (GenBank record OP007193) differed by one nucleotide in the 479 bp cyt b fragment from the lineage GAVIM01 (GenBank No. EF077166), which was detected in the closely related diver species, *Gavia immer* in North America. The novel Plasmodium lineage GAVSTE01 (GenBank record OP007192) differed by one nucleotide in the 479 bp cyt b fragment from a widespread lineage, TURDUS1 (GenBank record JN164734), that was previously found in a variety of European and Asian bird species from the families Fringilidae, Muscicapidae, Turdidae, Motacillidae, Paridae, Sylviidae, Accipitridae, Passeridae, Paridae, Hirundinidae, Sittidae, Scolopacidae and Certhiidae.

The other two Leucocytozoon lineages, each found in one individual, had exact matches (100%) to a lineage known from different European and Asian birds. The lineage CIAE02 is documented in Falconiformes, Gruiformes, Charadriiformes, Strigiformes, Piciformes, Ciconiiformes, Cuculiformes and Coraciiformes, and the lineage AMO02 in Falconiformes, Columbiformes, Piciformes and Passeriformes (Table 3).

### 3.3. Prevalence of Haemosporidian Parasites in Connection with Breeding Regions

**Figure 2:**
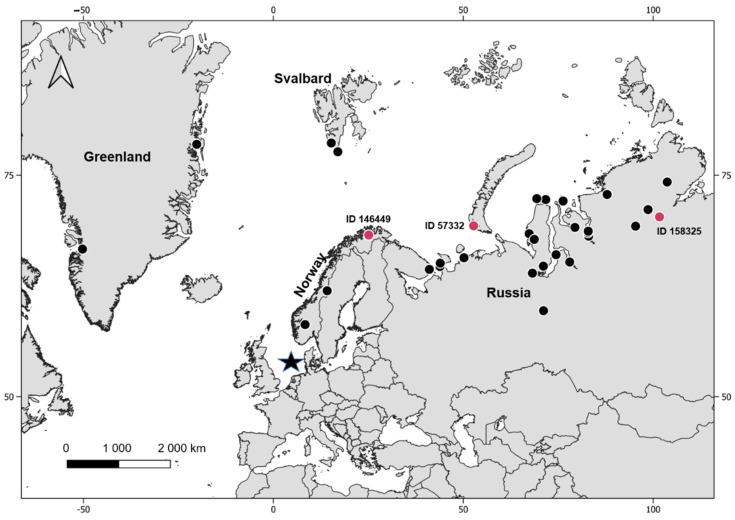
Overview of the breeding locations of the sampled red-throated divers. The capture area (sample location) is indicated by a black star, breeding locations of not infected individuals are indicated by black dots and breeding locations of infected individuals where a breeding location could have been assigned are indicated by red dots.

The breeding regions of red-throated divers in this data set were identified by additional analysis of tracking data and were located in Greenland, Svalbard, Norway and northern Russia, see Kleinschmidt et al. [40], (Figure 2). The breeding regions of two individuals, one (ID 146445) infected with *Leucocytozoon* and one (ID 146441) that showed a heterogenic infection with *Leucocytozoon* and *Plasmodium*, could not be assigned due to the early mortality of these individuals.

Individuals infected with haemosporidian parasites were detected in Norway and northern Russia, (Table 3, Figure 2). In Norway, one in three individuals was infected with the distinct novel Leucocytozoon lineage GAVSTE02, resulting in a prevalence of 33.0% (ID 146449, Table 3, Figure 2). In northern Russia 2 in 25 birds each had a single infection with Leucocytozoon (ID 158325, 57332, Table 3, Figure 2) resulting in a prevalence of 8.3%. One of these two Leucocytozoon infections was assigned to the lineage AEMO02 and a breeding site in Novaya Zemla (53°E, 71°N). The Leucocytozoon sequence found in the other individual that headed to the Taimyr peninsula (101°E,72°N) had an insufficient sequence length and no lineage could be identified. The birds analysed from breeding areas in Greenland (n = 2) and Svalbard (n = 2) tested negative.

## 4. Discussion

In this study we were able to define the presence–absence, prevalence and lineage richness of haemosporidian parasites in a previously unstudied species, the red-throated diver.

### 4.1. Presence–Absence of Haemosporida in Red-Throated Divers

The red-throated divers in our sample set could be assigned to the European breeding population [54] and the NW European wintering population [55]. The sampled birds were captured in the eastern German Bight (North Sea) and originated from Greenland, Svalbard, Norway and Arctic Russia. Of these breeding regions, we detected Haemosporida in individuals from Norway and Arctic Russia. No haemosporidian parasites were detected in individuals from Greenland and Svalbard. Admittedly, the sample size of birds from these regions in our sample set was small, with two birds per region, and reliable determination of presence–absence likely requires a larger sample size to detect a single Haemosporida. Little information is known so far about the presence of avian blood parasite infections in these regions, at least to our knowledge. Investigations in Greenland of gyrfalcons (*Falco rusticolus*) and peregrine falcons (*Falco peregrinus*) showed no presence of haemosporidian parasites [56], though potential vectors, such as black flies (*Prosimulium ursinum*) and mosquitos (*Aedes impiger*), were sampled. The absence of haemosporidian parasites was linked with temperature limitations and temperatures ranging from 0°–15° during July and August in Greenland [56]. In Svalbard little auks were sampled for haemosporidian parasites but these were not detected in the sampled birds [57]. Martinez et al. [58] screened samples from snow buntings from Svalbard for haemosporidian parasites but could not detect these parasites either. The absence of haemosporidian parasites in individuals from Svalbard and Greenland in our data set is in line with the general absence of haemosporidian parasites at these high arctic latitudes. In Norway, the presence of the haemosporidian parasites *Leucocytozoon* and *Plasmodium* was detected by Hellgren [59] in bluethroats (*Luscinia svecica*) over a wide geographic area and with various lineages. In other arctic regions the information about occurrence and distribution is fragmented. Information and positive detections in birds from Alaska has increased over the past years [10,60,61,62,63,64]. Recent studies investigating the Russian Arctic detected Haemosporida in passerine birds in southwestern Yamal (Russian Arctic), with a relatively high prevalence, with *Leucocytozoon* being the most prevalent compared to *Haemoproteus* and *Plasmodium* [65].

### 4.2. Haemosporidian Parasite Species and Lineage Diversity Detected in Red-Throated Divers

*Leucocytozoon* was the most abundant haemosporidian parasite in our data set. The more or less exclusive detection of *Leucocytozoon* might be related to the climatic conditions in arctic breeding locations. Oakgrove et al. [61] also showed for arctic regions in Alaska the highest prevalence of *Leucocytozoon* in birds across a latitudinal gradient. *Leucocytozoon* seems to have a higher cold tolerance than other haemosporidian parasites and is able to persist at high evaluations and in colder regions [66,67]. Furthermore, the high number of lineages found in arctic regions in Alaska suggests a potential adaptation in these arctic regions [62]. In the Russian Arctic, recent studies confirm the high prevalence of Leucocytozoon in passerines [65] and sea ducks [68]. Numerous studies suggest the relationship between temperature, seasonality and vegetation and the patterns of pathogen distribution [62,69,70], which might explain *Leucocytozoon* as the most prevalent and genetically diverse of haemosporidian parasites in Arctic regions.

*Haemoproteus* was absent from our dataset. The absence of *Haemoproteus* might be explained by a possible competitive exclusion between Leucocytozoon and Haemoproteus which has already been suggested by Oakgrove et al. [62,71].

*Plasmodium* was detected with a distinct novel lineage GAVSTE01 (GenBank record OP007192) in one individual which was co-infected with *Leucocytozoon*. The lower detection rate in our sample set compared to *Leucocytozoon* might be explained by climatic conditions in arctic breeding regions. *Plasmodium* prevalence is thought to be temperature dependent, which is an important factor in explaining variations in *Plasmodium* prevalence [72].

We found a high lineage diversity for *Leucocytozoon* with three lineages (CIAE02, GAVSTE02, AMO02, GenBank records KC962152, OP007193, LC440381) in our sample set considering five infected individuals and the total sample size (n = 45). The lineage diversity might be related to the geographical range among the breeding locations of infected individuals and a region-specific occurrence of vectors and parasites. Species of *Haemoproteus* and *Leucocytozoon* are described as being more specific and restricted to closely related species or the same family, unlike *Plasmodium* which can be considered as more host-generalised and is unlikely to be coevolved with vector species [7,73,74,75,76,77,78]. The distinct novel *Leucocytozoon* lineage GAVSTE02 in our dataset has a 1 bp difference from the *Leucocytozoon* lineage GAVIM01 that was previously found in *Gavia immer*, a closely related diver species. The *Leucocytozoon* lineage GAVIM01 is transferred by a highly exclusive relationship between a black fly, *Simulium annulus*, and *Gavia immer* [35,36,79]. Hellgren et al. [80] suggested that the association between blackfly species and host species hinders the transmission of parasites between different host-groups. Whether the novel *Leucocytozoon* lineage displays a similar exclusive relation to a specific vector and red-throated divers cannot be tested with our dataset.

### 4.3. Prevalence of Haemosporidian Parasites in Red-Throated Divers

The prevalence of all sampled red-throated divers screened in this dataset (n = 45) was 11.0%. The general low prevalence of seabirds documented in other studies [8,81,82] therefore also seems to apply for red-throated divers. Red-throated divers breed in arctic coastal inland habitats and outside of the breeding season this species prefers marine offshore habitats. Consequently, the transmission of haemosporidian parasites through vectors most likely occurs during the summer months which present the breeding period for these birds. Even during the breeding season, this species prefers coastal locations of nesting sites close to marine habitats [30,83,84]. Due to their preference for marine habitats, the transmission risk for blood parasites is rather low [85] and restricted to the breeding season. Considering a breeding region-specific prevalence and only individuals with a given breeding location, we found for individuals breeding in northern Russia a prevalence of 8.3% and for individuals breeding in Norway a comparatively high prevalence of 33.0%. Studies for sea ducks, which show a similar annual routine to red-throated divers, showed a similar low prevalence of, e.g., adult spectacled eiders (*Somateria fischeri*) in Alaska with 6.5% [10]. However, it must also be considered that the prevalence in our study may be underestimated as we sampled red-throated divers during the non-breeding season in winter and spring. Within the life cycles of the genera *Plasmodium, Haemoproteus* and *Leucocytozoon*, persistent stages are formed, which can remain latent in various tissues or organs of the host organisms and are thus largely protected from the host immune system [1,86]. These stages are thought to occur primarily when transmission cannot occur, such as during the winter months when vectors are generally unavailable [1,86]. Therefore, the prevalence in the blood might have been below the detection threshold of our methodology. Breeding origins were not identified for all infected individuals, due to the early mortality of two individuals after capture. Whether these early mortalities were linked to haemosporidian parasite infection and a possible cumulative effect of the capture-tagging procedure can neither be ruled out nor proven, as we had no chance to recover the dead bodies and to analyse blood smears or organs for increased parasitaemia. These early mortalities occurred in infected and not infected individuals in similar proportions (infected 8.8%; not infected 11.0%).

## 5. Conclusions

Our study presents a first proof of the two haemosporidian parasite genera *Leucocytozoon* and *Plasmodium* in red-throated divers, with the first record of a distinct novel lineage in each of the two parasite genera. Red-throated divers are increasingly affected by impairments to which they are sensitive, such as increasing anthropogenic activity in their stationary non-breeding habitats that have led to displacement effects [87]. A possible resulting increased stress level or poorer body condition might weaken the immune system, making the host more vulnerable to an outbreak of the disease. An infection with parasites in general, and haemosporidian parasites in particular, could cause a downturn/deterioration of the physical condition of a host. Here, the information about a prevalence of haemosporidian parasites provides important information to fully evaluate potential pre-loads. The red-throated diver is a migratory seabird species. How a haemosporidian infection affects migratory performance is not yet fully understood but, in general, migratory performance decreases with increasing infection intensity [29,88,89]

Since haemosporidian infection may have effects on reproductive success, condition, survival, host metabolism or migratory behavior, it may be considered a pre-load when evaluating the indirect and cumulative effects of anthropogenic or other stresses that result in an increased risk of suffering from elevated stress levels. However, in general, the low haemosporidian prevalence suggests a rather low importance of these parasites in this context. If only individuals from Norway are considered, however, the comparatively higher prevalence here might indicate a higher sensitivity of individuals from this breeding region.

## Figures and Tables

**Table 1:** Primer sequences used for the amplification of blood parasite DNA (Haemoproteus/Plasmodium/Leucocytozoon).

Primer	Primer Sequence	Target Dann
HaemNFI	5′-CATATATTAAGAGAAITATGGAG-3′	*Haemoproteus/Plasmodium/Leucocytozoon*
HaemNR3	5′-ATAGAAAGATAAGAAATACCATTC-3′	*Haemoproteus/Plasmodium/Leucocytozoon*
HaemF	5′-ATGGTGCTTTCGATATATGCATG-3′	*Haemoproteus/Plasmodium*
HaemR2	5′-GCATTATCTGGATGTGATAATGGT-3′	*Haemoproteus/Plasmodium*
HaemFL	5′-ATGGTGTTTTAGATACTTACATT-3′	*Leucocytozoon*
HaemR2L	5′-CATTATCTGGATGAGATAATGGIGC-3′	*Leucocytozoon*

**Table 2:** Reference sequences included in the molecular phylogenetic analysis.

Reference	Blood Parasite	MalAvi Lineage	Host and Country
JN164734	*Plasmodium circumflexum*	TURDUS1	*Sylvia atricapilla* (Spain)
AF495576	*Plasmodium circumflexum*	TURDUS1	*Turdus philomelos* (Sweden)
KC962152	*Leucocytozoon* sp.	CIAE02	*Buteo buteo* (Turkey)
EF607287	*Leucocytozoon* sp.	CIAE02	*Circus aeruginosus* (Germany)
EF077166	*Leucocytozoon* sp.	GAVIM01	*Gavia immer* (North America)

**Table 3:** Positively tested blood parasite infections in red-throated divers, with ID (ARGOS-ID/Bird-ID), breeding region, parasite genera and lineage. Novel lineages are indicated in bold.

ID (ARGOS ID)	Breeding Region	Blood Parasite Genus	Lineage
146441	n.a.	*Leucocytozoon*	CIAE02
**146445**	**n.a.**	***Leucocytozoon***	**GAVSTE02**
**146449**	**Norway**	***Leucocytozoon***	**GAVSTE02**
**146441**	**n.a.**	***Plasmodium***	**GAVSTE01**
158325	Arctic Russia	*Leucocytozoon*	n.a.
57332	Arctic Russia	*Leucocytozoon*	AMO02

## Data Availability

Newly generated sequences are available in GenBank: OP007192, OP007192. The hosts and sites table is available at the MalAvi database: http://mbio-serv2.mbioekol.lu.se/Malavi/.

## Appendix A. New Lineages Identified and Corresponding Requested GenBank Records Which Are Withheld and Will Be Added Officially after the Publication Is Accepted

**Table A1:** Specific information on the new lineages submitted to MalAvi.

Lineage Name	Sequence	GenBank Record	Parasite Genus	Host Species	Host Species ID
GAVSTE01	GCAACAGGTGCTTCATTTGTATTTATTCTAACTTATTTACATATTTTAAGAGGATTAAATTATTCATATTCATATTTACCTTTATCATGGATATCTGGATTACTTATATTCTTAATATCTATAGTTACAGCTTTTATGGGTTATGTATTACCTTGGGGTCAAATGAGTTTCTGGGGTGCCACTGTAATTACTAATCTATTATATTTTATACCTGGACTTGTTTCATGGATTTATGGTGGATATCTTGTAAGTGACCCAACATTAAAAAGATTCTTTGTATTACATTTTACATTTCCATTTATAGCTTTATGTATTGTATTTATACATATATTCTTTCTACATTTACAAGGTAGCACTAATCCTTTAGGGTATGATACAGCTTTAAAAATACCCTTCTATCCAAATCTTTTAAGTCTCGATATTAAAGGATTTAATAATGTATTAGTATTATTTTTAGCACAAAGTTTATTTGGAATATT	OP007192	*Plasmodium*	*Gavia stellata*	146441
GAVSTE02	TCAACAGGTGCATCATTTGTATTTATATTAACATACTTACATATCTTAAGAGGATTAAATTATTCTTTTACTTACTTACCTCTATCATGGATAAGTGGTTTAGCACTATTCTTAATATTTATTGTAACTGCTTTTATGGGTTATGTCTTACCATGGTGTCAAATGAGTTTTTGGGGAGCTACTGTTATCACTAATCTATTATATTTTATTCCTGGATTAATAAATTGGGTTTGTGGTGGATTTATTATCAATGACCCAACTCTAAAAAGATTCTTTGTATTACATTTTATATTCCCATTTGTAGCTCTAGCTATTGTATTTATACATATATTCTTCTTACATATTCAAGGTAGCACTAATCCTTTAGGGTATGATACACCTTTAAAAATACCATTCTATCCAAATCTATTAACTTTAGATGTTAAAGGATTTAATTATGTATTAGTATTATTCCTATTTCAAAGTTTATTTGGAATTGC	OP007193	*Leucocytozoon*	*Gavia stellata*	146445
GAVSTE02	TCAACAGGTGCATCATTTGTATTTATATTAACATACTTACATATCTTAAGAGGATTAAATTATTCTTTTACTTACTTACCTCTATCATGGATAAGTGGTTTAGCACTATTCTTAATATTTATTGTAACTGCTTTTATGGGTTATGTCTTACCATGGTGTCAAATGAGTTTTTGGGGAGCTACTGTTATCACTAATCTATTATATTTTATTCCTGGATTAATAAATTGGGTTTGTGGTGGATTTATTATCAATGACCCAACTCTAAAAAGATTCTTTGTATTACATTTTATATTCCCATTTGTAGCTCTAGCTATTGTATTTATACATATATTCTTCTTACATATTCAAGGTAGCACTAATCCTTTAGGGTATGATACACCTTTAAAAATACCATTCTATCCAAATCTATTAACTTTAGATGTTAAAGGATTTAATTATGTATTAGTATTATTCCTATTTCAAAGTTTATTTGGAATTGC	OP007194	*Leucocytozoon*	*Gavia stellata*	146449

**Table A2:** Specific information on the sample locations of birds infected with new lineages.

Host Species	Host Species ID	Host Status	Sample Location: Country	Sample Location: Region	Sample Location: Site Name	Sample Location: Latitude	Sample Location: Longitude	Sample Location: Altitude
*Gavia stellata*	146441	migratory	Germany	Schleswig Holstein	German Bight North Sea	54°47.028′	07°39.8262′	0
*Gavia stellata*	146445	migratory	Germany	Schleswig Holstein	German Bight North Sea	54°50.2569′	07°39.16704′	0
*Gavia stellata*	146449	migratory	Germany	Schleswig Holstein	German Bight North Sea	54°53.35668′	07°49.63914′	0

**Table A3:** List of positively tested red-throated divers for blood parasite infections, with ID (ARGOS-ID), parasite genera and identity match. Sample ID 158325 is only available with the forward sequence and therefore MalAvi hit and lineage information is not available.

ID (ARGOS ID)	Breeding Region	Blood Parasite Genus	Similarity GenBank	Closest Lineage (MalAvi)	MalAvi Hit (bp)
146441	n.a.	*Leucocytozoon*	100%	CIAE02	479/479
146445	n.a.	*Leucocytozoon*	99%	GAVIM01	474/475
146449	Norway	*Leucocytozoon*	99%	GAVIM01	474/475
146441	n.a.	*Plasmodium*	99%	TURDUS1	478/479
158325	Arctic Russia	*Leucocytozoon*	95%	n.a.	n.a.
57332	Arctic Russia	*Leucocytozoon*	100%	AMO02	479/479
